# RNA-based therapies for colorectal cancer: targeting the β-catenin pathway via microbiota -modulated miRNAs

**DOI:** 10.3389/fmolb.2025.1736094

**Published:** 2026-01-27

**Authors:** Rajkumar Prabhakaran, Ramkumar Muthu, Rajkumar Manickam, Venkatesh Subramanian, Karthikeyan Mahendran

**Affiliations:** 1 Department of Biochemistry, Karpagam Academy of Higher Education (Deemed to be University), Coimbatore, Tamil Nadu, India; 2 Center for Cancer Research, Karpagam Academy of Higher Education, Coimbatore, Tamil Nadu, India; 3 Department of Biotechnology, Sri Kaliswari College (Autonomous), Sivakasi, Tamil Nadu, India; 4 Department of Biotechnology, Karpagam Academy of Higher Education (Deemed to be University), Coimbatore, Tamil Nadu, India; 5 Department of Biotechnology, Manonmaniam Sundaranar University, Tirunelveli, Tamil Nadu, India; 6 Department of Microbiology, PSG College of Arts and Science, Coimbatore, Tamil Nadu, India

**Keywords:** beta-catenin signaling pathway, colorectal cancer, microbiota, microRNA, therapeutic efficacy

## Abstract

Colorectal cancer (CRC) is a significant problem with worldwide public health consequences. One key factor in the genesis and progression of CRC is the aberrant upregulation of the Wnt/β-catenin signaling pathway. Inhibitors designed to target β-catenin directly have not been effective in clinical trials, whereas miRNAs have been shown to regulate post-transcriptional components of the Wnt/β-catenin signaling pathway. Microorganisms in the gut also produce miRNAs that regulate CRC-related genes at the post-transcriptional level, including those involved in the Wnt pathway. An example is *Fusobacterium nucleatum*, which increases expression of the oncogenic miR-135b/miR-21, thereby inhibiting the expression of the tumor suppressors APC/PTEN and stabilizing β-catenin. This results in increased MYC expression. Another example is *Bacteroides fragilis*, which reduces miR-200c expression, thereby promoting epithelial-mesenchymal transition (EMT). However, this increase in EMT is countered by miR-145 and miR-203, which are upregulated by probiotic treatment, and these miRNAs inhibit the oncogenes *CTNNB1* and *LEF1*. There are currently several reviews that address subsets of the pathways involved in the dysregulation of β-catenin, as well as the therapeutic potential of miRNAs, and reviews that address microbiota interaction with CRC, but none that combine these elements within the framework of a mechanistic axis for the CRC microbe-miRNA-β-catenin-tumor phenotype, nor therapies based upon that axis/mechanism*.* This review examines the β-catenin signaling pathway in CRC and its regulation by miRNAs. It summarizes the roles of miRNAs in CRC, highlights oncogenic and tumor-suppressive miRNAs, and outlines specific miRNAs that are targets of the β-catenin pathway. It also covers microbiota-host interactions, including bidirectional links between gut microbes and miRNAs, effects on intestinal homeostasis, and microbial metabolites that alter miRNA expression. Recent advances in RNA-based therapeutic strategies and progress in clinical trials are included to frame the current translational relevance.

## Graphical Abstract

**Figure:**
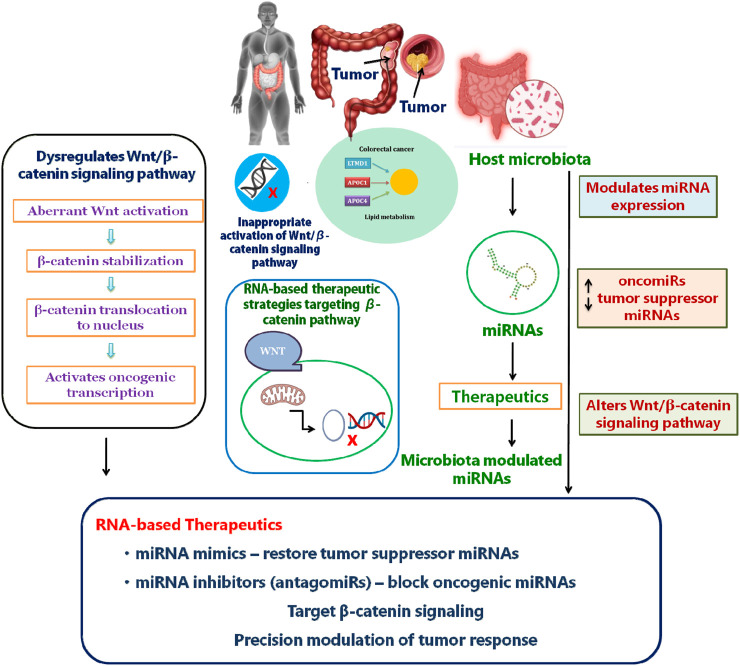

## Highlights

- CRC is a focal point of the typical dysregulation of the Wnt/β-catenin signaling pathway.
- RNA-based strategies (mimics, antagomirs, siRNAs, and aptamers) have therapeutic potential.
- The interplay between microbiota and miRNAs may lead to a precise therapeutic approach for CRC.
- This may also be a bottleneck for next-generation RNA therapeutics.
- The challenge was for targeting, delivery, and clinical translation of RNA-based strategies.

## 1 Introduction

Colorectal cancer (CRC) is considered one of the most prevalent and lethal cancers in the world. It is the second leading malignant cause of cancer-related deaths, and the third most diagnosed cancer (Zhang et al., 2025). Despite several significant strides in screening, diagnosis, and treatment, CRC is still a significant global public health burden, in part due to possible delays in starting treatment, high recurrence rates, and innate drug resistance to conventional treatment (Fadlallah et al., 2024). Thus, a proper understanding of the molecular mechanisms involved in CRC development is important to improving patient outcomes and developing new therapeutic targets. One of the principal molecular features of CRC is deregulated Wnt/β-catenin signaling that drives tumor initiation, metastasis, and progression (Zeng et al., 2025).

Aberrant β-catenin activation results in dysregulated proliferation, maintenance of cancer stemness, and enhanced cellular survival, which makes it a compelling therapeutic target. Direct targeting of β-catenin has not been clinically successful; therefore, targeting additional regulators may be beneficial. It has recently been shown that microRNAs (miRNAs) are significant post-transcriptional regulators of β-catenin signaling (Lei et al., 2020; Sevim et al., 2025). miRNAs can function as oncogenic drivers or tumor suppressors; they also regulate β-catenin stability and downstream factor expression. The simultaneous coordination of multiple signaling pathways creates opportunities for miRNAs to inform therapeutic innovation and biomarker discovery in CRC management. Additionally, the gut microbiome, a complex community of microorganisms residing in the gut, is recognized as an important modulator of host gene expression, including microRNA (miRNA) expression (Manzat-Saplacan et al., 2015).

Bacteria-derived metabolites and signaling molecules can also alter miRNA expression and, most importantly, modify β-catenin signaling, cancer localization, and treatment response. The interplay among the microbiome, miRNAs, and the β-catenin signaling system constitutes a unique therapeutic axis for precision medicine in colorectal cancer (Yuan et al., 2019; Xing et al., 2022).

### 1.1 Microbiota contributes to therapeutic response

A modified microbiota may alter biological equilibrium by regulating physiological status through metabolites, genes, and proteins. Recent investigations of the microbiota have reported its involvement in diverse diseases that influence the microbiota-gut axis, as well as the microbiota-lung, liver, brain, bone, and vascular axes. Altered conditions can provoke diseases such as cognitive impairment, allergy, autoimmunity, obesity, diabetes, inflammatory bowel disease, and cancer. The studies from Aguilar et al. (2019) and Zhou et al. (2022) reported that the function of miRNAs mediates the communication between intestinal microbiota and host intestinal epithelial cells (Aguilar et al., 2019). Li et al. (2020) reported on the interaction between the gut microbiota and miRNAs and their role in host pathophysiology, including intestinal, neurological, cardiovascular, and immune health. Allegra et al. (2020) outlined that the microbiota shapes miRNA activity, and these interactions influence cancer development. Zhou et al. (2022) summarize evidence that microbiota-related mechanisms influence CRC.

### 1.2 RNA-based therapies

Several factors have led to the establishment of a bidirectional relationship between the gut microbiota and miRNA expression. A large body of evidence supports this conclusion, as analyses of 76 differentially expressed miRNAs from colorectal tissues of CRC patients have identified correlations with specific bacterial taxa (*Fusobacterium, Akkermansia,* and *Roseburia*) (Yuan et al., 2018). In another example, specific co-expression pairs involving many bacterial genera (most prominently *Porphyromonas* and *Bifidobacterium*) and miRNAs were observed in metastatic CRC (Zhou et al., 2022). In addition, butyrate appears to be the key metabolite that mediates the effect of gut microbiota on miRNAs (especially MYC-inhibition-induced oncogenesis of the miR-17∼92 cluster) (Yuan et al., 2018). However, while β-catenin appears to play an important role in linking microbiota and Wnt/β-catenin signaling, there is limited direct evidence supporting this connection among the three components of the gut microbiota-miRNA-β-catenin axis (Yuan et al., 2018). Only one study reports a direct relationship between *Fusobacterium* nucleatum and Wnt/β-catenin signaling, mediated by FadA binding to E-cadherin and FadA-mediated downregulation of GSK3β, thereby increasing β-catenin levels (Pös et al., 2023). Several studies indicate that miR-21 regulates Wnt/β-catenin signaling by targeting PTEN, PDCD4, and DKK2 (Pös et al., 2023). Functionally, the three classes of miRNAs regulate cell growth, proliferation, migration, and innate immunity, as well as chemotherapeutic resistance. Correlation is seen between all three components of the gut microbiota-miRNA-β-catenin axis. However, there is no direct evidence supporting a relationship between the gut microbiota and β-catenin in the four studies that examined these variables (Zhou et al., 2022). Therefore, although some data indicate bidirectional interactions between the gut microbiota and miRNA, and between miRNA and β-catenin, a complete characterization of the pathways linking these three components remains to be investigated.

### 1.3 Knowledge gap statement

Existing studies describe microbiota or miRNA biology in broad terms, but they rarely integrate microbe-miRNA-β-catenin interactions into a single mechanistic axis. They do not map how specific organisms drive defined miRNA changes that activate or repress beta-catenin and shape CRC behavior. This gap limits the development of RNA-based treatments that depend on microbiota status. RNA therapeutics need a clear mechanistic framework to guide patient selection and predict response. Microbiota composition influences miRNA profiles that regulate β-catenin, which means microbial status may determine whether RNA-based therapies succeed. This review explains the mechanistic links between microbes, miRNA, and beta-catenin. It summarizes CRC-focused evidence, highlights regulatory axes that drive tumor growth or suppression, and outlines therapeutic opportunities at this interface.

## 2 β-catenin signaling pathway in CRC

**FIGURE 1:**
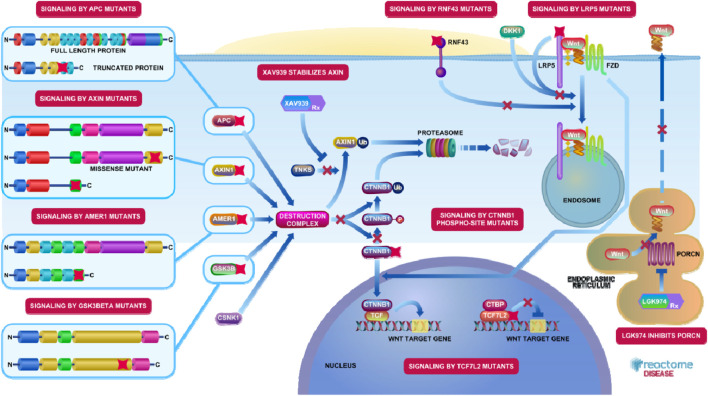
The Wnt signaling pathway was first linked to cancer when the Wnt1 gene was found to be activated by the mouse mammary tumor virus in tumor development. Aberrant Wnt/β-catenin activation occurs in approximately 90% of sporadic cases of CRC, and it has also been associated with other solid tumors, including kidney, liver, and gastric cancer. Loss-of-function mutations within genes belonging to the destruction complex (APC, AXIN, and AMER1), or gain-of-function mutations in CTNNB1, resulted in constitutive signaling. Both the canonical and non-canonical pathways of Wnt pathways are involved in tumor growth, invasiveness, and ultimately, metastasis (Reactome Pathway ID: R-HSA-4791275).

In the absence of Wnt ligands, the canonical Wnt/β-catenin pathway controls intestinal epithelial homeostasis by regulating β-catenin degradation through a destruction complex containing APC, AXIN, GSK3β, and CK1 (Figure 1). This complex phosphorylates cytoplasmic β-catenin, targeting it for ubiquitination and proteasomal degradation. Wnt ligand binding to Frizzled/LRP co-receptors inhibits the destruction complex, stabilizing β-catenin, which translocates to the nucleus, binds TCF/LEF transcription factors, and activates gene programs controlling proliferation and stemness (He and Gan, 2023). In normal colorectal epithelium, this balance ensures controlled renewal, but sustained β-catenin activation drives oncogenic proliferation and tumor initiation. Mutations that result in the loss of function of *adenomatous polyposis coli* (APC) and those that activate *CTNNB1* disrupt β-catenin degradation (Wang et al., 2025). This disruption leads to its accumulation in the nucleus, thereby promoting the continuous expression of oncogenic targets such as MYC and cyclin D1. These genetic alterations represent key regulatory checkpoints for β-catenin stabilization and are promising targets for RNA-based therapies aimed at restoring degradation or blocking downstream effectors. Epigenetic mechanisms, such as promoter hypermethylation or histone modifications, also contribute to β-catenin pathway hyperactivation by silencing Wnt antagonists (e.g., SFRPs, DKKs), thereby promoting sustained signaling (Kafle et al., 2024). These epigenetic changes can be modulated by RNA-based approaches to reactivate pathway inhibitors.

Post-transcriptional regulation by miRNAs, long non-coding RNAs (lncRNAs), and RNA-binding proteins provides dynamic control over β-catenin signaling in CRC, and these represent accessible therapeutic nodes. Post-transcriptional regulation of β-catenin signaling in CRC involves specific miRNAs and lncRNAs that have shown therapeutic potential. For instance, members of the miR-200 family, such as miR-200c, suppress β-catenin signaling by targeting ZEB1 and other EMT regulators, thereby decreasing tumor invasiveness and stemness (Longo et al., 2025). miR-34a, a tumor-suppressive miRNA, directly targets β-catenin mRNA (*CTNNB1*) and downstream effectors, reducing proliferation and metastasis. Conversely, oncogenic miRNAs, such as miR-21 and miR-135b, enhance β-catenin activity by repressing negative regulators, including APC and DKKs (Lei et al., 2020). LncRNAs also modulate β-catenin signaling. For example, lncRNA CCAT1 acts as a competing endogenous RNA (ceRNA) sponge for tumor-suppressive miRNAs that target β-catenin pathway components, thereby increasing pathway activation and tumor progression (Lei et al., 2024). lncRNA H19 promotes β-catenin nuclear translocation and activation by directly binding β-catenin, creating a feedforward loop that sustains oncogenic transcription programs. Targeting such lncRNAs using antisense oligonucleotides or siRNAs offers a promising therapeutic avenue. These RNA modulators represent critical checkpoints at which intervention can restore the balance of β-catenin signaling. Therapeutic delivery of tumor-suppressive miRNAs or inhibitors of oncogenic miRNAs/lncRNAs via nanoparticles, viral vectors, or other nanocarriers holds significant promise for overcoming resistance and targeting CRC more effectively (Song et al., 2024). Integrating knowledge of specific RNA regulators with patient molecular profiles could enable personalized RNA-based therapies targeting β-catenin-driven CRC (Voutsadakis, 2025; Yan et al., 2025).

## 3 miRNAs and β-catenin pathway regulation

miRNAs are short, non-coding RNA molecules ranging from 18-25 nucleotides in length that are essential for regulating gene expression at the post-transcriptional level. They achieve this by attaching to the 3′ untranslated regions (UTRs) of the target mRNA. miRNAs may inhibit translation or promote degradation, thereby regulating numerous important cellular processes, including cell growth, programmed cell death, and cell movement. Moreover, miRNA dysregulation can contribute to cancer development, including CRC, functioning as either an oncogene or tumor suppressor, depending on the message and context (Ali Syeda et al., 2020). miRNAs directly linked to β-catenin regulation in CRC was summarized in Table 1.

**TABLE 1:** miRNAs directly linked to β-catenin regulation in CRC.

miRNA	Class	Direct beta-catenin target	Evidence strength	Model type
miR-552	Oncogenic	APC	Strong	Cell lines, clinical tissues (Zou et al., 2020)
miR-483	Oncogenic	Indirect via PTEN, SMAD4	Strong	Cell lines, clinical tissues (Zhou et al., 2021)
miR-671-5p	Oncogenic	AXIN2, GSK3beta	Moderate-strong	Cell lines, clinical tissues (Shirzad et al., 2025)
miR-146a	Tumor suppressor	Indirect (NF-kB, EGFR)	Moderate-strong	Cell lines, animal (Farc et al., 2023)
miR-29a	Tumor suppressor	beta-catenin mRNA	Moderate	Cell lines, clinical tissues (Farc et al., 2023)
miR-215-5p	Tumor suppressor	Indirect via HOXB9, epiregulin	Strong	Cell lines, clinical tissues (Vychytilova-Faltejskova et al., 2017)
miR-144-3p	Tumor suppressor	beta-catenin, TCF4	Strong	Cell lines, clinical tissues (Yang et al., 2022)
miR-203	Tumor suppressor	beta-catenin, ZEB1, ZEB2	Strong	Cell lines, clinical tissues (Yang et al., 2022)
miR-377-3p	Tumor suppressor	beta-catenin coactivators	Moderate	Cell lines, clinical tissues (Yang et al., 2022)
miR-532-3p	Tumor suppressor	FZD7, LRP6	Strong	Cell lines, animal (Yang et al., 2022)
miR-346-5p	Tumor suppressor	beta-catenin, ZEB1, ZEB2	Moderate	Cell lines (Yang et al., 2022)
miR-519d-3p	Tumor suppressor	DVL2, GSK3beta	Moderate	Cell lines, animal (Shirzad et al., 2025)
miR-103/107	Oncogenic	Axin2	Strong	Cell lines, clinical datasets (Chen et al., 2019)
miR-520e	Tumor suppressor	Indirect via AEG-1	Moderate	Cell lines, animal (Zhao et al., 2022)
miR-188-5p	Oncogenic	Indirect via Ras	Moderate	Cell lines, clinical tissues (Pająk et al., 2025)
miR-217	Oncogenic	TCF7L2	Moderate	Cell lines (Zhao et al., 2022)
miR-143	Tumor suppressor	KRAS, beta-catenin	Moderate	Cell lines, animal (Pająk et al., 2025)
miR-411	Oncogenic	DKK1	Weak-moderate	Cell lines (Zhao et al., 2022)
miR-1205	Oncogenic	SFRP1	Weak-moderate	Cell lines (Zhao et al., 2022)
miR-125b	Tumor suppressor	beta-catenin mRNA	Moderate	Cell lines, clinical tissues (Pająk et al., 2025)
miR-452-5p	Tumor suppressor	beta-catenin mRNA	Moderate	Cell lines (Koyama et al., 2023)

### 3.1 Overview of microRNAs and their role in cancer

**FIGURE 2:**
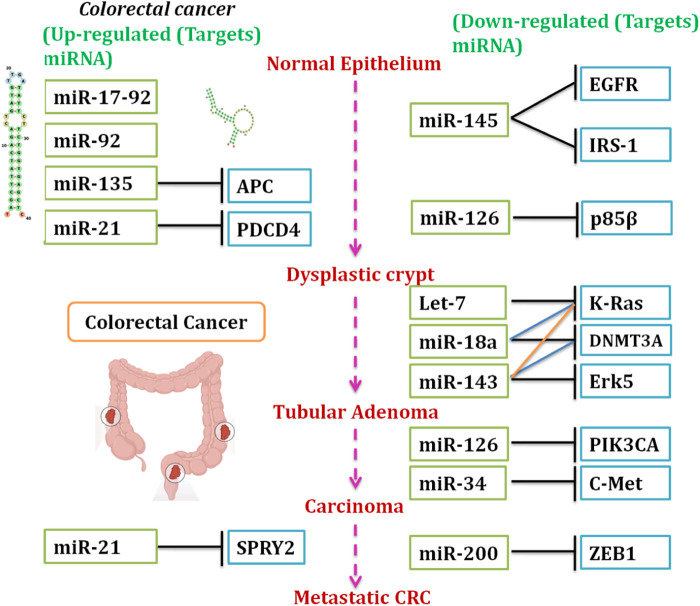
Abnormal miRNA levels are commonly observed during CRC development. The schematic depicts (upregulated; downregulated) miRNAs and pathways modulated by miRNAs at ordered tumor stage progression. In CRC (upper panel), aberrant expression of specific miRNAs, including miR-17-92, miR-92, miR-135, and miR-21, advances the progression of normal epithelium to carcinoma by modulating pathways that target APC, PDCD4, and SPRY2. In contrast, downregulated miRNAs, including miR-145, miR-126, and miR-200, modulate pathways targeting EGFR, IRS-1, p53, and ZEB1, thereby promoting metastasis. The dotted arrows indicate the trajectory of ordered tumorigenesis from normal to invasive/metastatic (https://www.kegg.jp/pathway/map05206).

**FIGURE 3:**
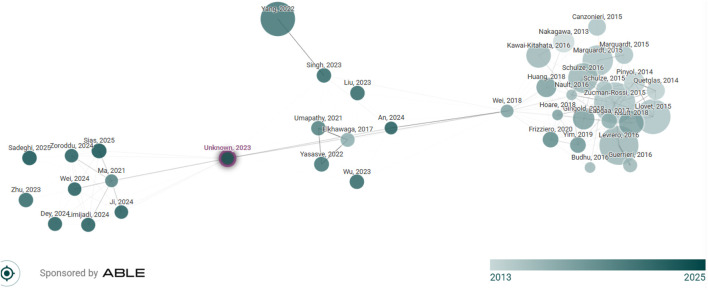
Network map of papers on MicroRNAs and the β-catenin Pathway. Data derived from connected papers (original search term: “MicroRNAs and β-catenin Pathway Regulation”). Node size indicates citation count, node color indicates publication year. (Each node represents an academic paper related to the original paper. Papers are arranged according to their similarity (this is not a citation tree), node size is the number of citations, node color is the publishing year, and similar papers have strong connecting lines and cluster together). Source: Connected Papers (www.connectedpapers.com).

MiRNAs are evolutionarily conserved classes of RNAs that regulate numerous biological processes, including cell differentiation, proliferation, apoptosis, and immune responses (Huang et al., 2010). In cancer, miRNAs can act as oncogenes (oncomiRs) or tumor suppressors, depending on their target genes (Figure 2). miRNA dysregulation has been implicated in the initiation, progression, and metastasis of cancer and in resistance to therapy in a multitude of cancers. In particular, miRNAs regulate key components of Wnt/β-catenin signaling, a signaling pathway that is dysregulated in a variety of human cancers, including CRC (Wu et al., 2019; Hamidi et al., 2025). Figure 3 shows the “MicroRNAs and β-catenin Pathway Regulation” related article search using connected papers.

### 3.2 Oncogenic miRNAs in CRC

#### 3.2.1 miR-501-3p in CRC progression

miR-501-3p has been recognized as an oncogenic miRNA in CRC. Higher levels of miR-501-3p expression are found in CRC tumor tissues than in adjacent normal tissues. This miRNA promotes CRC development by activating the Wnt/β-catenin signaling pathway. Downregulation of miR-501-3p in CRC has been shown to inhibit cell proliferation, induce G1 cell cycle arrest, and suppress sphere formation in CRC cells (Wu et al., 2019).

#### 3.2.2 miR-183 and inhibition of Wnt pathway inhibitors

miR-183 has also been studied in cancer as a direct inhibitor of Wnt and downstream pathway inhibitors, including AXIN1, AXIN2, and GSK3β, thereby enhancing canonical Wnt signaling activated by miR-183. This may also contribute to oncogenesis in CRC (Song et al., 2024; Gupta et al., 2025).

### 3.3 Tumor-suppressive miRNAs in CRC

#### 3.3.1 miR-142-3p and β-catenin suppression

In CRC, miR-142-3p acts as a tumor suppressor by directly targeting β-catenin mRNA of (CTNNB1). Overexpression of miR-142-3p reduces β-catenin levels, prevents its accumulation in the nucleus, and inhibits the transcriptional activity of β-catenin. This results in reduced proliferation and colony formation in CRC cells. Importantly, mutations in miR-142-3p binding sites on β-catenin mRNA abolished this inhibitory effect (Liu et al., 2021).

#### 3.3.2 miR-200 family and EMT regulation

The miR-200 family, comprising miR-200a, miR-200b, and miR-200c, is a known inhibitor of epithelial-to-mesenchymal transition (EMT). Specifically, miR-200a targets ZEB1 and ZEB2, which repress E-cadherin expression, thereby increasing E-cadherin levels and enhancing cell-cell adhesion. The increase in E-cadherin, a cell adhesion molecule, reduces the levels of free β-catenin in the cytosol and inhibits Wnt/β-catenin signaling. Dysregulation of the miR-200 family is associated with increased EMT and metastasis in CRC (Huang et al., 2010).

#### 3.3.3 miR-101 and mutual inhibition with Wnt pathway

miR-101 and the Wnt pathway have a mutually suppressive relationship in CRC. Overexpression of miR-101 significantly reduces β-catenin accumulation in the nucleus, whereas activation of the Wnt pathway inhibits the expression of miR-101. This feedback loop emphasizes the careful modulation of β-catenin by miR-101 in CRC (Peng et al., 2016).

### 3.4 Specific miRNAs targeting β-catenin pathway

#### 3.4.1 miR-199a-5p and Wnt pathway inhibition

miR-199a-5p suppresses the Wnt/β-catenin signaling pathway by directly targeting multiple signaling factors including FZD4, JAG1, and Wnt2. This ultimately increases AXIN2 expression and inhibits several downstream target genes, including *MYC* and *CCND1*. In CRC, miR-199a-5p acts as a tumor suppressor by inhibiting β-catenin activity (Peng et al., 2016).

#### 3.4.2 miR-384-5p and myocardial fibrosis

miR-384-5p inhibits Wnt/β-catenin signaling by targeting FZD1, FZD2, TGRBR1, and LRP6, thereby reducing β-catenin phosphorylation (Liu et al., 2022).

#### 3.4.3 miR-214 and EZH2 interaction

miR-214 binds to β-catenin and EZH2, a histone methyltransferase, to inhibit Wnt/β-catenin signaling. This dual targeting action makes miR-214 a strong tumor suppressor in hepatocellular carcinoma and potentially in CRC (Peng et al., 2016).

#### 3.4.4 miR-320 and FOXM1 regulation

miR-320 targets β-catenin and the transcription factor, *FOXM1*, which are involved in cell proliferation. By targeting these genes, miR-320 inhibits Wnt/β-catenin signaling and tumor growth in prostate and CRC (Peng et al., 2016).

## 4 Gut microbiota and miRNA regulation

The gut microbiota, a diverse community of microorganisms found in the gastrointestinal tract, is critically important to human health (Table 2, 3). Additionally, gut microbiota and host miRNA interactions are important for various physiological (pathological) system functions. miRNAs are short, non-coding RNA molecules that act by binding to mRNAs to silence or alter gene expression at the post-transcriptional level to generate proteins. The purpose of the current study was to present three aspects of gut microbiota and miRNA regulation: (A) microbiota-host interactions, (B) microbial metabolites altering miRNA gene expression, and (C) signaling molecules generated from the microbiota affecting miRNAs. Bidirectional communication between the gut microbiota and miRNAs is an emerging area of research with applications to diseases such as obesity, cancer, cardiovascular disease, and neurodegeneration. The present study analyzed all three aspects of miRNA regulation of the gut microbiota, drawing on the latest preclinical advances.

**TABLE 2:** Below are CRC-related miRNAs influenced by microbes, and their validated gene targets.

miRNA	Regulated by microbe	β-Catenin target/Effector	Tumor phenotype	References
miR-21	*Fusobacterium nucleatum↑*	PTEN↓, β-catenin↑	Proliferation, chemoresistance	Sadeghloo et al. (2025)
miR-155	*E. coli(pks+) ↑*	SOCS1↓, β-catenin/STAT3↑	Inflammation→tumorigenesis	Sadeghi et al. (2025), Yang et al. (2021)
miR-200c	*Bacteroides fragilis↓*	ZEB1↑, β-catenin nuclear↑	EMT, metastasis	Chang et al. (2025), Guo et al. (2025)
miR-34a	*Enterococcus faecalis↓*	CTNNB1↓, LEF1↓	Stemness suppression	Kunte et al. (2011), Li et al. (2021)
miR-135b	*F. nucleatum↑*	APC↓, β-catenin↑	Wnt hyperactivation	Zeng et al. (2024)
miR-124	*Dysbiosis ↓*	CDK6↓, β-catenin↓	Cell cycle arrest	Chen et al. (2025)
miR-29a	*F. nucleatum ↑*	DNMT3B↓, SFRP2↑, β-catenin↓	Tumor suppression	Guz et al. (2021), Rubinstein et al. (2013), Triantaphyllopoulos et al. (2025)
miR-145	*Lactobacillus ↑*	β-catenin, c-Myc↓	Stemness↓	Torres-Maravilla et al. (2021), Yamada et al. (2013)
miR-375	*F. nucleatum ↑*	TCF4↓, Wnt targets↓	Invasion↓	Sadeghloo et al. (2025), Xu et al. (2016)
miR-31a	*F. nucleatum ↑*	RNF43↓, β-catenin↑	Stemness↑	Tang et al. (2023)
miR-18a	*Lactobacillus acidophilus and Bifidobacterium bifidum ↓*	PTEN inhibition enhances PI3K-Akt- β-catenin crosstalk	Tumor progression↑	Heydari et al. (2019)

**TABLE 3:** Bacterial taxa associated with colorectal cancer.

Bacterial taxa	Associated with CRC	miRNA correlations	Metabolites
*Fusobacterium nucleatum*	Increased risk of CRC, especially in the early and metastatic stages	Correlated with cancer-related pathways; modulates miRNA levels	Not specified
*Bacteroides fragilis*	Higher abundance in CRC	Modulates miRNA expression	Not specified
*Porphyromonas*	Associated with most miRNAs	Co-expression with multiple miRNAs	Not specified
*Bifidobacterium spp.*	Associated with most miRNAs	Co-expression pairs identified	Not specified
*Faecalibacterium prausnitzii*	Decreased in CRC	Associated with miRNA changes	Butyrate production
*Akkermansia*	Altered in CRC	Correlated with CRC pathway miRNAs	Not specified
*Roseburia*	Reduced abundance	Correlated with cancer pathways	Butyrate

### 4.1 Microbiota-host interactions

#### 4.1.1 Bidirectional communication between gut microbiota and miRNAs

**FIGURE 4:**
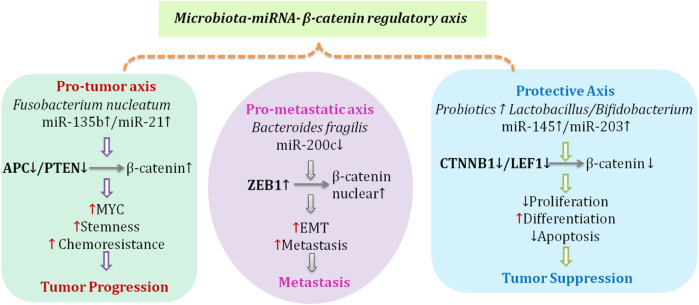
Microbiota-miRNA-β-catenin regulatory axes in colorectal cancer.

There is bidirectional interaction between the gut microbiota and miRNAs. miRNAs derived from the host can modify the presence or activity of the gut microbiota, and can also influence host miRNA expression (Figure 4). For example, host miRNAs can be incorporated into extracellular vesicles and transported into the gut lumen, where they may be taken up by particular bacterial species, potentially affecting microbial growth or gene expression. Both miR-200b-3p and miR-7704 were associated with differences in gut microbiota composition in colitis and chronic hepatitis B (Cuinat et al., 2025). In addition, metabolites and vesicles from the gut microbiota may alter host miRNAs and influence host physiology. This means that the interactions discussed here demonstrate the complexity of host-microbiota interactions and their importance for the maintenance of intestinal homeostasis.

#### 4.1.2 Role in intestinal homeostasis

Intestinal homeostasis is co-regulated by the gut microbiota and miRNAs, which act together through the gastrointestinal barrier, immune responses, and microbiome diversity. miR-143 and miR-150, for example, have been reported to promote gut barrier function and restore microbiota composition in colitis models (Ayyanar and Vijayan, 2025). miRNAs, such as miR-223 and miR-155, also exert functional immune regulation by modulating target genes involved in antigen presentation and inflammation. Disruption of microbiota-host interactions can lead to dysbiosis, or imbalances in microbial flora, which are associated with disease states such as inflammatory bowel disease (IBD), obesity, and cancer. The miRNAs miR-130b-3p, miR-185-5p, and miR-21-5p, for example, exhibit distinct expression profiles between healthy lean and sick obese individuals and correlate with the bacterial species *Bacteroides eggerthii* and *Dorea longicatena*, which are involved in metabolic control (Drago et al., 2025).

A consistent finding across studies was altered microbial composition in CRC, characterized by shifts from Firmicutes to Bacteroidetes and reduced abundance of butyrate-producing bacteria (Yuan and Subramanian, 2019; Yuan et al., 2018). Sixteen miRNA-genus co-expression pairs, comprising eight microbial genera and 15 miRNAs, were identified in metastatic CRC (Zhou et al., 2022). The has-miR-3943 was targeted by most microbial genera (Zhou et al., 2022). Short-chain fatty acids, particularly butyrate, have emerged as key mediators linking the microbiota to host cell regulation. Butyrate provides approximately 70% of the energy needs of colonic epithelial cells (Yuan and Subramanian, 2019), and its reduction in CRC patients corresponds with decreased SCFA levels and butyrate-producing bacteria. Additional metabolites mentioned include acetate and propionate.

### 4.2 Microbial metabolites influencing miRNA expression

#### 4.2.1 Microbial metabolites as regulators of miRNAs

Gut microbiota generates diverse metabolites that can regulate miRNA expression in the host, including short-chain fatty acids (SCFAs), lipopolysaccharides (LPS), and secondary bile acids. These metabolites serve as signaling molecules that can affect gene expression in host cells. For example, SCFAs (butyrate and propionate) have been shown to regulate miRNAs that modulate inflammation and metabolism (Cuinat et al., 2025). Another example is the genotoxic compound colibactin, produced by *Escherichia coli* strains, which induces miR-20a-5p expression and promotes colon tumor growth by secreting growth factors (Dalmasso et al., 2014). These illustrations highlight the potential influence of microbial metabolites on miRNA regulatory networks in both health and disease.

#### 4.2.2 Implications for disease

These illustrations highlight the potential influence of microbial metabolites on miRNA regulatory networks in both health and disease. The relationship between microbial metabolites and miRNAs has potential consequences on the prevention and treatment of disease. For example, both lipopolysaccharide (LPS) and short-chain fatty acids (SCFAs), produced by the gut microbiota, can significantly affect the expression of miRNAs involved in glucose and lipid metabolism and, consequently, metabolic homeostasis (Ionescu et al., 2022). Referring to cancer, microbial metabolites (*i.e*., secondary bile acids) can alter levels of miRNA expression, ultimately affecting tumor progression and drug resistance. For instance, miRNAs deregulated in cancer-associated signaling pathways can affect tumor initiation and progression, as well as drug resistance to chemotherapy (Nikolaieva et al., 2022).

### 4.3 Signaling molecules from microbiota affecting miRNAs

#### 4.3.1 Extracellular vesicles and miRNA regulation

Gut microbiota-derived extracellular vesicles (EVs) harbor numerous RNA families, including miRNAs, that may influence gene expression in the host. EVs can cross the intestinal barrier to deliver miRNAs to host cells, thereby promoting inflammation, metabolism, and immune responses (Du et al., 2021). For example, *Fusobacterium* nucleatum-derived EVs have been shown to deliver miRNAs that promote CRC by targeting host genes involved in immune evasion and apoptosis (Nikolaieva et al., 2022).

#### 4.3.2 Lipopolysaccharides and immune modulation

LPS is an important part of the outer membrane of Gram-negative bacteria, and is another signaling molecule that alters miRNA expression. LPS stimulates TLR4 signaling pathways in host cells, thereby inducing the expression of miRNAs associated with inflammatory responses. For example, miR-155 is upregulated in response to LPS stimulation and promotes the production of pro-inflammatory cytokines (Kurowska-Stolarska et al., 2011).

#### 4.3.3 Therapeutic potential

Microbial signaling molecules can also modulate miRNA expression, providing a potential opportunity for therapeutic approaches (Table 4). For example, probiotics that alter the composition of the gut microbiota while producing beneficial metabolites are associated with miRNA expression modulation, offering a new avenue for managing diseases such as IBD, obesity, and cancer, whose pathways are linked to altered miRNA expression. Some studies have demonstrated that *Lactobacillus fermentum* and *Bifidobacterium animalis* can enhance the expression and/or function of miRNAs implicated in gut barrier integrity and immune modulation (Ayyanar and Vijayan, 2025).

**TABLE 4:** Selected publications (2013–2025) investigating miRNA-mediated regulation of the β-catenin signaling pathway and related therapeutic approaches in CRC and other solid tumors.

Title	Authors	Year
MicroRNAs in the regulation of Wnt/beta-Catenin, NF-kB, PI3K/AKT, STAT3, p53, and Hedgehog pathway (Tufail, 2023)	Muhammad Tufail	2023
Genomics Studies in Hepatocellular Carcinoma via Next-Generation Sequencing (Wei et al., 2017)	Xiyang Wei, Niya Liu, X. Wang, Junfang Ji	2018
Bioinformatics Analysis of LINC00917 Targets miR-3690/DDX39A Axis to Exacerbate Pancreatic Cancer Cell Growth Using Real-Time Quantitative Reverse Transcription PCR (An et al., 2024 )	Baiping An, Yi Cai, Jie Zhu, Yuan Liu	2024
Radiation Therapy for Chondrosarcoma (Ma et al., 2021)	T. Ma, T. Delaney, A. Kalbasi	2021
Pharmacological targeting of P300/CBP reveals EWS: FLI1-mediated senescence evasion in Ewing sarcoma (Wei et al., 2024)	Wei Erdong, Ana Mitanoska, Quinn O'Brien, Kendall Porter, MacKenzie Molina, Haseeb Ahsan, Usuk Jung, Lauren Mills, Michael Kyba, D. Bosnakovski	2024
Identification of 3-Aryl-1-benzotriazole-1-yl-acrylonitrile as a Microtubule-Targeting Agent (MTA) in Solid Tumors (Zoroddu et al., 2024)	Stefano Zoroddu, L. Sanna, Valentina Bordoni, Lyu Weidong, S. Gadau, Antonio Carta, David J. Kelvin, L. Bagella	2024
Untangling the Role of MYC in Sarcomas and Its Potential as a Promising Therapeutic Target (Sias et al., 2025)	Fabio Sias, Stefano Zoroddu, Rossana Migheli, L. Bagella	2025
Role of Molecular Targeted Therapeutic Drugs in Treatment of Glioblastoma: A Review Article (Singh, 2023)	Singh Himanshu	2023
Clinical significance of TNFAIP3 rs2230926 T > G gene polymorphism in Egyptian cases with rheumatoid arthritis (Elkhawaga et al., 2017)	Samy Y Elkhawaga, A. Abulsoud, Mostafa M. El-Shafey, M. M. Elsayed	2017
Advances in genomic hepatocellular carcinoma research (Huang et al., 2018)	Weitai Huang, A. Skanderup, Caroline G. Lee	2018
Current issues in genomic heterogeneity in hepatocellular carcinoma and their implications for clinical practice (Schulze and Zucman-Rossi, 2015)	Schulze, K., and Zucman-Rossi, J	2015
Transcriptional expression of miRNAs under glucose depletion/2-deoxy-d-glucose in HCC: A possible genetic footprint of angiogenesis and its hallmarks ( Umapathy et al., 2021 )	Devan Umapathy, Manochitra Karthikeyan, Kumar Ponnuchamy, Antony Joseph Velanganni Arockiam	2021
Role of dysregulated miRNA profiles as hallmarks in the pathogenesis and differential regulation of tongue squamous cell carcinoma (Yasasve and Saravanan, 2022)	M. Yasasve, M. Saravanan	2022
Genetic Landscape and Biomarkers of Hepatocellular Carcinoma (Zucman-Rossi et al., 2015)	Zucman-Rossi, J., Villanueva, A., Nault, J. C., and Llovet, J. M	2015
Glioma targeted therapy: insight into the future of molecular approaches (Yang et al., 2022)	Yang Keyang, Zhijing Wu, Hao Zhang, Nan Zhang, Wantao Wu, Zeyu Wang, Ziyu Dai, Xun Zhang, Liyang Zhang, Yu Peng, Weijie Ye, Wenjing Zeng, Zhixiong Liu, Q. Cheng	2022
CircSMAD2 accelerates endometrial cancer cell proliferation and metastasis by regulating the miR-1277-5p/MFGE8 axis (Wu et al., 2023)	Yuehua Wu, Fuhua Wang, Jing Shi, Xiangyun Guo, Feng Li	2023
The role of miR-16 and miR-34a family in the regulation of cancers: A review (Sadeghi et al., 2025)	Zahra Sadeghi, Mehrnoush Malekzadeh, Mohammad Sharifi, B. Hashemibeni	2025
Upregulation of MAD2L1 mediated by ncRNA axis is associated with poor prognosis and tumor immune infiltration in hepatocellular carcinoma: A review ( Liu et al., 2023 )	Sizhe Liu, Mingsan Miao, Le Kang	2023
Molecular Profiling of Human Hepatocellular Carcinoma (Budhu and Wang, 2016)	A. Budhu, X. Wang	2016
Genomic Signatures of Risk Factors and Molecular Identification of HCC Subtypes (Nault and Zucman-Rossi, 2016)	Nault, J. C., and Zucman-Rossi, J	2016
Mechanisms of HBV-induced hepatocellular carcinoma (Levrero and Zucman-Rossi, 2016)	Levrero, M., and Zucman-Rossi, J	2016
Hepatocellular carcinoma: an overview of clinico-pathological and molecular perspectives (Canzonieri et al., 2015)	V. Canzonieri, L. Alessandrini, L. Caggiari, T. Perin, M. Berretta, R. Cannizzaro, RE V.DE	2015
Mechanisms of Disease: The Damaged Genome in HCC (Hoare, 2018)	M. Hoare	2018
Genetics of Hepatocellular Carcinoma: Risk Stratification, Clinical Outcome, and Implications for Therapy (Labgaa et al., 2017)	Labgaa, S. Torrecilla, Iris Martinez-Quetglas, D. Sia	2017
Genetic profiling of hepatocellular carcinoma using next-generation sequencing (Schulze et al., 2016)	Schulze K, J. Nault, A. Villanueva	2016
Integration of genomic information in the clinical management of HCC (Quetglas et al., 2014)	Quetglas I, Agrin Moeini, R. Pinyol, J. Llovet	2014
Current Translational and Clinical Challenges in Advanced Hepatocellular Carcinoma. (Frizziero et al., 2020)	M. Frizziero, M. McNamara, A. Lamarca, R. Pihlak, Roopa Kurup, R. Hubner	2020
Pathobiology of Hepatitis B Virus-Induced Carcinogenesis (Guerrieri et al., 2016)	F. Guerrieri, L. Belloni, N. Pediconi, M. Levrero	2016
The role of molecular enrichment on future therapies in hepatocellular carcinoma (Nault et al., 2018)	J. Nault, P. Galle, J. Marquardt	2018
The Genomic Landscape and Its Clinical Implications in Hepatocellular Carcinoma (Yim and Lee, 2019)	S. Yim, Ju-Seog Lee	2019
Comprehensive analyses of mutations and hepatitis B virus integration in hepatocellular carcinoma with clinicopathological features (Kawai-Kitahata et al., 2016)	Kawai-Kitahata, F., Asahina, Y., Tanaka, S., Kakinuma, S., Murakawa, M., Nitta, S., and Watanabe, M.	2016
Molecular Profiling of Liver Tumors: Classification and Clinical Translation for Decision Making (Pinyol et al., 2014)	Pinyol, R., Nault, J. C., Quetglas, I. M., Zucman-Rossi, J., and Llovet, J. M	2014
Advances in targeted therapies for hepatocellular carcinoma in the genomic era (Llovet et al., 2015)	J. Llovet, A. Villanueva, A. Lachenmayer, R. Finn	2015

##### 4.3.3.1 Recent RNA-based therapy clinical trial advancements

RNA-based therapeutics is an emerging class of cancer therapies. It provides several strategies and mechanisms for regulating gene expression via mRNA, siRNA, miRNA, antisense oligonucleotides (ASOs), and RNA aptamers. For CRC, no identified trial combines an RNA-based platform with direct targeting of the β-catenin pathway. Recent clinical progress needs to be integrated. Phase 1 clinical trial of early RNA therapies, such as MRX34 (miR-34A mimic), have demonstrated targeted binding in solid tumors. New research programs include antagomirs targeting miR-21 and miR-135b, siRNAs targeting CTNNB1, and CRISPR-Cas13 systems designed to degrade beta-catenin transcripts. Trials using lipid nanoparticle siRNAs against Wnt pathway nodes in gastrointestinal cancers provide relevant examples-the two RNA-based therapies, autogene cevumeran and pelareorep, employ fundamentally different mechanisms.

Autogene cevumeran is a personalized mRNA vaccine approach that generates immune responses against patient-specific neoantigens (Kopetz et al., 2022a). In contrast, pelareorep exploits selective viral replication in KRAS-mutated cells to induce cytotoxic T-cell activation (Goel et al., 2020). Neither specifically modulates β-catenin signaling. Conversely, the two β-catenin/Wnt pathway-targeted therapies, RXC004 and FOG-001, are small-molecule inhibitors rather than RNA-based platforms. RXC004 acts upstream by inhibiting Porcupine to block Wnt ligand secretion (Kopetz et al., 2022a), while FOG-001 directly blocks β-catenin: TCF transcriptional activity at the most downstream pathway node (Papadopoulos et al., 2024). The positioning of FOG-001 as “first-in-class” (Papadopoulos et al., 2024) highlights the novelty of direct β-catenin targeting, which may eventually create opportunities for combination with RNA-based immunotherapies. Patient selection biomarkers differ substantially across trials. The Wnt pathway-targeted trials select patients based on pathway-activating mutations (RNF43, RSPO fusions, APC, or β-catenin mutations) (Kopetz et al., 2022a). At the same time, pelareorep requires KRAS mutations (Goel et al., 2020), and autogene cevumeran requires ctDNA positivity and identifiable neoantigens (Kopetz et al., 2022b). All colorectal cancer trials focusing on metastatic disease require microsatellite-stable (MSS) status (Kopetz et al., 2022a), thereby targeting a population with limited immunotherapy options.

The combination of RXC004 with nivolumab (Kopetz et al., 2022a) and the demonstrated immune activation by pelareorep suggest emerging interest in combining pathway-targeted and immunomodulatory approaches. However, direct integration of RNA-based platforms with β-catenin inhibition remains unexplored in the current clinical trial landscape.

### 4.4 Delivery mechanisms for RNA-based therapies

#### 4.4.1 Extracellular vesicles (EVs)

Extracellular vesicles, including exosomes, occur naturally and have the potential to deliver RNA-based therapeutics. EVs have numerous advantages, such as biocompatibility, low immunogenicity, and the ability to cross biological barriers. EVs are engineered to carry siRNA or miRNA and target specific cancer cells through surface ligand modifications. Clinical studies have shown variable efficacy of EV-derived RNA biomarkers in CRC detection (Datta et al., 2022). miR-1246 and miR-23a were evaluated in 88 patients and showed sensitivities of 95.5% and 92%, respectively, and specificities of 84% and 81%, respectively (Zou et al., 2019). miR-17-3p and miR-92a were tested in 170 and 196 patients and showed moderate sensitivities of 64 percent and 84 percent, with specificities of 70 percent and 71.2 percent, respectively (Huang et al., 2010). Let-7a and miR-223 showed limited diagnostic value, with sensitivities close to 50 percent and specificities ranging from 41 percent to 44 percent (Ng et al., 2009). In a cohort of 315 patients, miR-135 showed 95% specificity but 46.2% sensitivity (Ogata-Kawata et al., 2014). miR-29a showed a better balance in 196 patients, with a sensitivity of 69% and a specificity of 89.1%. miR-1224-5p and miR-1229 exhibited low sensitivity and specificity, ranging from 20% to 31.8% (Ogata-Kawata et al., 2014). The miR-17-92 cluster demonstrated moderate performance in 316 patients, with 69.5% sensitivity and 81.5% specificity (Kral et al., 2018).

##### 4.4.1.1 Application in CRC

Folate-displaying EVs: In a patient-derived xenograft (PDX) mouse model of CRC, siRNA could be delivered using folate-modified EVs, according to a study. During weeks 4 and 5, the treatment resulted in a significant reduction in tumor size and weight compared to controls, with p-values of 0.0098 and 0.0387 at each week (Pi et al., 2018).

Reprogrammed EVs: Native EVs can now be reprogrammed using RNA nanotechnology for siRNA delivery. In three animal models, these EVs demonstrated enhanced cancer cell-specific targeting and efficient intracellular siRNA release, which resulted in tumor suppression.

##### 4.4.1.2 Advantages and challenges

EVs provide nuclease protection and targeted biodistribution, improving the stability and efficacy of RNA therapeutics. However, challenges such as low loading efficiency, scalability, and potential off-target effects remain to be addressed.

#### 4.4.2 Nanoparticles

Nanoparticles are synthetic carriers that can encapsulate RNA molecules, protecting them from enzymatic degradation and enhancing their delivery to target cells. Various types of nanoparticles, including lipid nanoparticles (LNPs) and polymer-based nanoparticles, have been developed for RNA delivery.

##### 4.4.2.1 Applications in CRC

LNPs: Dicer-substrate siRNA (DsiRNA) targeting CTNNB1, formulated as DCR-BCAT in lipid nanoparticles, has shown promising results in preclinical models. In a CRC liver metastasis model, it reduced CTNNB1 mRNA levels by more than 50% and improved survival (Fujita and Demizu, 2025).

Smart nanoparticles: Nanoparticles engineered with tumor-specific ligands and stimuli-responsive properties have been used to enhance RNAi therapy. For example, liposomal platforms that co-deliver STAT3 siRNA and lidocaine achieved tumor control, highlighting their efficacy in CRC (Ren et al., 2025).

##### 4.4.2.2 Advantages and challenges

Nanoparticles offer precise targeting, prolonged circulation, and enhanced intracellular delivery. However, issues such as immunogenicity, toxicity, and high production costs need to be addressed for clinical translation.

#### 4.4.3 Viral vectors

Viral vectors, such as adenoviruses and lentiviruses, are widely used for gene delivery due to their high transduction efficiency. They can be engineered to deliver RNA-based therapeutics, including siRNA and miRNA, to cancer cells.

##### 4.4.3.1 Applications in CRC

- Oncolytic viruses: Oncolytic viruses, which selectively infect and kill tumor cells, have been combined with RNA-based therapies to enhance their efficacy. For instance, oncolytic reovirus sensitized microsatellite-stable (MSS) CRC to immune checkpoint inhibitors, such as anti-PD-1 therapy (Pérez-Domínguez et al., 2025).
- Adenoviral vectors: Adenoviruses have been used to deliver tumor antigens and siRNA, modulating the tumor microenvironment to enhance immunotherapeutic outcomes (Li et al., 2024).

##### 4.4.3.2 Advantages and challenges

Viral vectors provide efficient gene delivery and long-lasting expression of therapeutic RNA. However, concerns about immunogenicity, vector-specific neutralizing antibodies, and systemic clearance limit their clinical use.

### 4.5 Synergistic approaches in CRC therapy

Combining RNA-based therapies with conventional treatments, such as chemotherapy and immunotherapy, has shown synergistic effects in CRC. For example:

- siRNA and 5-fluorouracil (5-FU): A novel siRNA delivery system targeting beta-catenin demonstrated a synergistic effect with 5-FU in CRC cells, enhancing therapeutic efficacy (Ghareeb et al., 2025).
- siRNA and immunotherapy: DCR-BCAT, combined with PD-1 blockade, led to complete tumor regression and increased CD8^+^ T-cell infiltration in preclinical models (Fujita and Demizu, 2025).

These approaches highlight the potential of RNA-based therapies to enhance the efficacy of existing treatments and overcome resistance mechanisms in CRC.

### 4.6 Toxicity and off-target activity in miRNA therapeutics

Toxicity and off-target activity remain central obstacles in miRNA therapy (Segal and Slack, 2020; Wen et al., 2015). miRNAs regulate multiple transcripts; therefore, introducing mimics or inhibitors can disrupt pathways beyond the intended target (Wang et al., 2021). This produces unintended effects on cell growth, apoptosis, and immune signaling. Higher doses increase the risk of immune stimulation through Toll-like receptor activation. Chemical modifications improve stability but introduce new safety concerns related to hepatic and renal clearance. Delivery vehicles, such as lipid nanoparticles, can induce inflammation, activate the complement system, or accumulate in off-target organs. Variability in endogenous miRNA networks across patients further complicates the prediction of adverse effects. These toxicity risks require careful sequence design, dose control, and tissue-specific delivery (Ruan et al., 2025).

### 4.7 Translation challenges and future perspective

Translation of RNA-based therapeutics for CRC faces several hurdles. Delivery to colorectal tissue remains inefficient, as current carriers exhibit poor stability, low retention, and limited penetration. Off-target activity is every day, as miRNAs and siRNAs affect multiple transcripts. Patient-specific differences in gut microbiota composition alter miRNA responses and reduce reproducibility across studies. Redundant signaling within the Wnt and β-catenin networks also limits the impact of single-target RNA agents. Long-term safety data for repeated dosing in the gastrointestinal environment are limited, and there are concerns about inflammation and immune activation. Biomarker gaps persist due to the lack of a validated microbiota or miRNA signature to guide patient selection. Manufacturing quality, sequence consistency, and batch consistency remain regulatory expectations that slow translation.

Future advances are likely to come from engineered probiotics that deliver miRNA mimics or inhibitors to the colon, improved nanoparticles with colonic-targeted absorption, and integration of microbial and tumor miRNA profiles for personalized therapy. Combining RNA agents with small-molecule Wnt inhibitors, immunotherapy, or microbial modulators is expected to improve pathway suppression. Computational design tools that predict target networks will support the safe development of arrays. Early clinical trials that stratify patients based on microbiome composition and β-catenin activity will refine treatment selection and support progress toward clinical application.

## 5 Conclusion

Colorectal cancer remains a significant global health concern, with aberrant activation of the Wnt/β-catenin signaling pathway recognized as a major driver of tumor initiation, progression, and therapeutic resistance. Emerging evidence indicates that microbiota-regulated miRNAs can negatively modulate β-catenin activity, presenting new opportunities for the development of RNA-based therapeutic interventions. Depending on the pathways they target, miRNAs may act as oncogenic regulators (oncomiRs) or tumor suppressors.

Tumor-suppressive miRNAs such as miR-142-3p, the miR-200 family, and miR-34 inhibit β-catenin activation by directly targeting β-catenin mRNA or upstream modulators of the pathway. This inhibition leads to decreased cell proliferation, reduced epithelial-to-mesenchymal transition (EMT), and diminished metastatic potential. Beyond their regulatory role in signaling, miRNAs also intersect with lipid metabolic pathways-an important characteristic of colorectal cancer-by influencing genes such as APOC1 and INSL5. This convergence of signaling and metabolic regulation underscores the potential of miRNAs as candidates for combination RNA-based therapeutic strategies targeting both oncogenic pathways and metabolic dysregulation.

Future research should prioritize validating these mechanistic insights through comprehensive multi-omics approaches and rigorously designed human studies.
